# Immunotherapy in Hepatocellular Carcinoma: Is There a Light at the End of the Tunnel?

**DOI:** 10.3390/cancers11081078

**Published:** 2019-07-30

**Authors:** Amit Mahipal, Sri Harsha Tella, Anuhya Kommalapati, Alexander Lim, Richard Kim

**Affiliations:** 1 Department of Medical Oncology, Mayo Clinic, Rochester, MN 55906, USA; 2 Department of Internal Medicine, University of South Carolina School of Medicine, Columbia, SC 29209, USA; 3 Department of Gastrointestinal Oncology, H. Lee Moffitt Cancer Center, Tampa, FL 33612, USA

**Keywords:** liver cancer, immunotherapy, nivolumab, lenvatinib, sorafenib

## Abstract

Hepatocellular carcinoma (HCC) is the most common primary liver cancer with dismal prognosis when diagnosed at advanced stages. Surgical resection of the primary tumor or orthotropic liver transplantation serves as a potential curative option. However, this approach is highly dependent on the hepatic reserve and baseline functional status of the patient. Liver directed therapies such as portal vein embolization (PVE), trans-arterial chemoembolization (TACE), and systemic chemotherapy are employed in non-surgical candidates. Sorafenib was the only approved systemic therapeutic agent for almost a decade until the recent approval of lenvatinib by the United States Food and Drug Administration (FDA) as an alternate first-line agent. Regorafenib, nivolumab, pembrolizumab and cabozantinib are approved by the FDA as second-line agents in patients who failed or could not tolerate sorafenib. Ramucirumab was recently FDA approved for the subset of patients that have high alfa-fetoprotein levels (>400 ng/mL). A better understanding of tumorigenesis and encouraging clinical trial results that evaluated immune-checkpoint inhibitors opened doors for immunotherapy in HCC. Immune checkpoint inhibitors have demonstrated a prolonged median overall and progression-free survival in a subset of patients with HCC. On-going translational and clinical research will hopefully provide us with a better understanding of tumor markers, genetic aberrations and other factors that determine the immunotherapy response in HCC. In this review, we sought to summarize the potential role and future directions of immunotherapy in the management of HCC.

## 1. Introduction

Hepatocellular carcinoma (HCC) is the third-highest cause of cancer-related deaths globally, with a heterogeneous prognosis depending on the etiology, geographical location, and stage of the disease at presentation [1]. Incidence of the disease varies by geographical location with the majority of cases reported in the Asia-Pacific and sub-Sahara African region [2]. Risk factors, more prevalent in these regions, are known to be hepatocytic, thus leading to chronic inflammation, remodeling, and transformation of hepatic tissue if left untreated [3]. These risk factors include certain viral or parasitic infections, metabolic factors including obesity, and exposures to aflatoxins, alcohol, or tobacco. Chronic inflammation promotes cirrhosis and HCC development by causing an immune-suppressive microenvironment. The majority of patients with HCC have underlying cirrhosis at the time of initial diagnosis. Vaccination against Hepatitis B and C; treatment of active viral infections, and close monitoring with serial imaging studies and alfa-fetoprotein (AFP) levels have resulted in a decreasing incidence of HCC in Southeast Asia. There is however a rising incidence of non-alcoholic fatty liver disease (NAFLD), which has led to a higher incidence of HCC and its related mortality in other regions of the world such as the United States and Central Europe [4,5,6].

Therapeutic options for HCC are primarily based on the Barcelona-Clinic Liver Cancer system and Child-Pugh score. Surgical resection of the primary tumor is generally preferred for patients with localized disease. In patients who are not surgical candidates, liver-directed therapies such as portal vein embolization (PVE), radio-frequency ablation (RFE), stereotactic body radiation therapy (SBRT) and trans-arterial chemoembolization (TACE) are considered [2]. Orthotopic liver transplantation is a reasonable option in patients with Child-Pugh class B and C, or in patients who could not undergo surgical resection of the primary tumor [2]. In the United States, the general practice is that patients with HCC are evaluated for orthotropic liver transplantation based on the Milan criteria, which includes patients with a solitary tumor <5 cm, or 3 individual tumors each measuring <3 cm in maximum diameter, and without macro-vascular invasion or extra-hepatic extension. In patients that do not meet the Milan criteria at first evaluation, liver directed therapies (RFE, PVE, SBRT, and TACE) may be employed for down-staging the tumor depending upon the clinical condition [2]. These patients may later be re-evaluated for liver transplantation.

In patients with progressive disease after liver directed therapy and in patients with extra-hepatic extension or involvement of portal vein, systemic therapy is recommended if the liver function and performance status are adequate. After the approval of sorafenib (based on the SHARP trial) by the United States Food and Drug Administration (FDA) [7], there was a sad-saga of a decade where no targeted therapy or chemotherapy demonstrated survival advantage. However, as our understanding of the HCC tumorigenesis evolved, there has been a tremendous development in systemic therapies. In 2018, lenvatinib was approved by the FDA for patients with advanced HCC based on a global, phase III randomized, open-label non-inferiority trial when it was compared to sorafenib [8]. In this study, there was no overall survival (OS) difference between sorafenib and lenvatinib, however, lenvatinib had a better safety profile. Two other targeted-therapies regorafenib and cabozantinib were also FDA approved in 2017 and 2019, respectively, as second-line agents in patients with advanced HCC who either progressed or could not tolerate sorafenib [9,10]. Most recently, a vascular endothelial growth factor (VEGF) receptor-2 targeted monoclonal antibody, ramucirumab was FDA approved for the subset of patients that have high alfa-fetoprotein levels (>400 ng/mL) [11].

After the disappointing results with cytokines (interferon alpha-2b, interleukin-12) [12,13], a better understanding of HCC tumorigenesis and encouraging results were seen with nivolumab opened doors for immunotherapy in HCC [14]. While pembrolizumab showed encouraging results in a phase II trial [15], these results were not replicated with statistical significance in the phase III trial. In short, immune checkpoint inhibitors, including nivolumab and pembrolizumab, prolong median OS and progression-free survival (PFS), but only in a subset of patients with HCC. In addition, immunotherapy is currently being evaluated in combination with liver-directed therapies and as adjuvant therapy. On-going translational and clinical research will hopefully provide us a better understanding on tumor markers, genetic aberrations and other factors that determine the immunotherapy response in HCC. In this review, we sought to summarize both the tumorigenesis of HCC in brief, and the potential role and future directions of immunotherapy in the management of HCC.

## 2. HCC Tumorigenesis

HCC is a heterogeneous tumor both clinically and histo-pathologically. The majority of HCC cases are preceded by liver cirrhosis and focal areas of low- and high-grade dysplastic lesions, which results from chronic inflammation and fibrosis. Presence of stromal invasion differentiates HCC from dysplastic lesions. Next-generation sequencing has shown that the tumors harbor 20–100 mutations per genome on average [16]. The number of mutations in HCC depends on the etiological factors: tumors resulting from hepatitis B virus harbor a relatively higher number of mutations as the virus integrates into the human genome and replicates through virus reverse transcriptase. The virus also results in chromosomal instability due to an increased number of deletions and translocations. Hepatitis C virus, on the other hand, causes multiple deoxyribonucleic acid (DNA) breaks without integrating into the human genome. *ARID1A/D2* (chromatic remodeling pathway genes), *TP53*, *BCL-6*, *CTNNB1*, immunoglobulin genes, genes involved in Janus Kinase-Signal transducers and activators of transcription (JAK/STAT), and mitogen-activated protein kinase (RAS/MAPK) pathways are primarily targeted by the hepatitis C virus [17]. *TP53* mutations were seen in 18–50% of HCCs with the highest percentage seen in the geographic areas with a high percentage of hepatitis C cases [18,19,20]. *TP53* mutations were also implicated in fungal aflatoxin exposure [21]. Telomerase reverse transcriptase (TERT) was seen in 30–60% of HCCs and molecular studies have shown that genetic aberrations in TERT lead to premature liver fibrosis [22].

In addition to the gene mutations, epigenetic modifications such as DNA methylation, histone modification, chromatic remodeling (especially in genes *ARID1A*, *ARID1B*, and *ARID2*), copy number variations, and gene rearrangements have been implicated in the tumorigenesis of HCC [23]. Molecular studies have identified methylation defects (hypo or hypermethylation) in various genes such as *RASSF1A*, *SOCS-3*, *CDKNA2*, *MGMT*, and *GSTP1* [24,25,26,27]. Hepatitis B and C virus were known to cause dysregulation of DNA methylation and the hepatitis C virus was particularly implicated in methylation gene defects in Wnt pathways [28,29,30]. Moreover, the Wnt pathway is activated by hepatitis C virus proteins including NS3 and NS5, which leads to alterations in micro RNA-155 expressions and increasing tumor necrosis factor-alpha (TNF-α) levels. Mitogen-activated Protein Kinase pathway (RAS/MAPK) pathway activation was shown to be present in about 50% of HCC tumors. Phosphorylation of fibroblast growth factor (FGF), hepatocyte growth factor (HGF) pathway, and c-Met all lead to activation of the RAS/MAPK pathways that promote HCC tumorigenesis [31]. Multi-kinase inhibitors (Regorafenib, cabozantinib, and ramucirumab) that target these pathways were recently approved by the United States Food and Drug Administration (US FDA) for the management of HCC that are refractory to sorafenib. In addition, Lenvatinib, another tyrosine kinase inhibitor was approved by the FDA as a first-line agent in the management of HCC.

Apart from the mutations in the above described regulatory cell cycle pathways, HCC tissues were found to harbor a higher percentage of circulating regulatory T-cells and myeloid-derived suppressor cells (MDSCs) implicating their potential role in HCC tumorigenesis [32]. In addition, given the higher degree of antigen exposure from the gastrointestinal tract through the portal vein, an immune-suppressive environment is created by immune-suppressive cytokines such as interleukin-4, 5, 8 and 10. Moreover, immune-activating cytokines such as interleukin 1, TNF, and interferon-gamma are suppressed [33]. While all these factors promote HCC tumorigenesis, the tumor cells were shown to create an immune-suppressive tumor microenvironment by the programmed cell death (PD) pathway, which causes apoptosis of CD8+T-cells [34].

In summary, irrespective of the etiological factors, the final common pathway of HCC tumorigenesis is constant liver cell injury resulting in a vicious cycle of cell death, regeneration, and proliferation ultimately resulting in genomic instability, finally leading to HCC. Furthermore, given the continuous exposure to antigens through the portal vein blood supply, liver-intrinsic mechanisms create an immunosuppressed environment in the liver [35]. This “escape” from an immunologic environment results from an inhibition of arginase-1 and galectin-9 and increased expression of checkpoints that also promotes the tumorigenesis in HCC [36].

### Immune Tolerance and Chronic Necroinflammation in HCC Tumorigenesis

Hepatic tissue is constantly exposed to numerous toxins and antigens and it has intrinsic tolerance and immune escape mechanisms to prevent auto-immune destruction of the tissue. This immunosuppression is partly achieved by inhibitory cytokines interleukin-4, 5, 8, 10 and tumor growth factor-β released by Kupffer cells and endothelial cells of the liver [33]. In addition, decreased expression of surface molecules, CD80 and CD86 on the liver sinusoidal cells limit the activation of CD4+ T-cells [37]. Furthermore, programmed death ligand 1/2 (PD-L1/L2) that is expressed on kupffer cells, sinusoidal endothelial cells, hepatocytes, and stellate cells induce T-cell apoptosis thereby contributing to immune tolerance mechanism in the hepatic tissue [38]. Previous studies showed that the expression of PD-L1 increases during chronic viral infection and other inflammatory disorders of the liver, which in turn leads to tolerance to HCC tumor-associated antigens potentiating the HCC tumorigenesis. The chronic inflammatory state was further shown to be associated with augmented regulatory T-cell numbers, altered check-point expression and dendritic cell function, which inhibits immune attack on the infected hepatocytes [39]. Moreover, increased expression and upregulation of PD-1 was shown to be associated with progression of HBV-related hepatic cirrhosis to HCC and recurrence after surgical resection of the primary tumor [34,40]. Given the increased expression of PD-1/PD-L1 in chronic necro-inflammatory conditions and HCC tumorigenesis, PD-1/PD-L1 inhibitors evolved as therapeutic agents in the management of HCC.

## 3. Immune Landscape and Immunotherapy in HCC

Immunotherapy with cytokines (interferon alpha-2b, interleukin-12) has been evaluated in the management of HCC since the early 1990s with unsatisfactory results [12,13]. On the contrary, immunotherapy using anti-PD-1/PD-L1 agents and anti-cytotoxic T-lymphocyte associated protein-4 (CTLA-4) have shown promising and encouraging results in other malignancies including melanoma, lung cancer, renal cell carcinoma, and bladder cancer [41]. Checkpoint molecules such as CTLA-4, PD-1, and T cell immunoglobulin- and mucin-domain-containing molecule-3 (TIM-3) are expressed on T cells to prevent the exaggerated immune response. Cancer cells also create a tumor microenvironment that simulates checkpoint molecules thereby blocking the cytotoxic T-cells immune response towards cancer cells. As detailed in the tumorigenesis section, the PD-pathway (PD-1/PD-L1) has been implicated in HCC tumorigenesis and their expression correlated with higher post-surgical tumor recurrences. Pre-clinical studies have shown that overexpression of PD-L1 in HCC cell lines contributed to sorafenib resistance [42]. Increased regulatory T-cells and MDSC expression in HCC tissues were shown to be associated with aggressive tumors and poor outcomes [43]. Moreover, the opposing roles of key cytokines such as tumor growth factor-β render immunotherapy a challenge in the management of HCC [33]. However, immune checkpoint inhibitors act by blocking the tumor microenvironment, which suppresses cytotoxic T-cells, thereby exaggerating T-cell mediated cancer cell death. In the following sections, we discuss in detail the various immunotherapies evaluated in the management of HCC.

## 4. Cytokines

INF-α and IL-12 were the immunotherapeutic agents evaluated in HCC in the 1990s. Though, theoretically, INF-α should show promising results in HCC, the drug showed partial response only in 6% of the study subjects (two out of 30) and the majority of the patients discontinued the medication due to intolerable side effects [12]. Similarly, IL-12 did not yield promising results when evaluated in advanced gastrointestinal tumors including pancreatic, colon and HCC. Though post-therapy intra-tumoral evaluation showed an increased number of CD8+ T-lymphocytes, none of the HCC patients had promising tumor response rates [13,44].

Another cytokine, transforming growth factor (TGF)-β, was evaluated in HCC with encouraging results. TGF-β was shown to play a role in HCC tumorigenesis through constant hepatic remodeling and fibrosis. Moreover, TGF-β has been implicated in signaling pathways including Wnt, NOTCH, and MAPK that are known to play a key role in HCC tumorigenesis [45]. Galunisertib (LY2157299) is a TGF-β inhibitor that was evaluated in patients with HCC that progressed on sorafenib (or who did not tolerate sorafenib) in a phase II trial. The cohort with elevated AFP levels >200 ng/mL had a median OS of 8.4 months compared to 17 months in patients with AFP levels <200 ng/mL. Interestingly, patients who achieved a >20% reduction in TGF-β1 levels had a significant improvement in OS. [46,47]. Galunisertib is currently being evaluated in the management of HCC in several ongoing clinical trials either alone (NCT02240433, NCT01246986, NCT02178358) or as a combination therapy (NCT02906397, NCT02423343).

## 5. Vaccines in HCC Management

Peptide vaccines against glypican-3 and oncolytic and dendritic cell vaccines have been evaluated in the management of HCC. Earlier case series that evaluated the potential role of pulsed dendritic vaccines against AFP and its peptides showed mixed results [48]. Out of the two patients evaluated, one patient who had T-cell immunity against AFP had marginal benefit in PFS. Later, a phase I trial from Japan evaluated the role of anti-tumor vaccine using AFP-derived peptides in 15 patients with HCC [49]. The study showed that the AFP-peptide vaccine was well-tolerated while resulting in a complete response in one patient and stable disease in eight patients. The patient who had complete response had AFP-peptide derived T-cell receptor induction against the tumor cells. 

Carcinoembryonic antigen glypican-3 is another HCC specific antigen, which has been implicated in HCC carcinogenesis through Wnt signaling pathway. Glypican-3 peptide intradermal vaccine was evaluated in a phase I trial involving 33 patients with HCC. Of these 33 patients, 19 patients had stable disease while one patient had a partial response [50]. The study showed that the glypican-3 vaccine is effective in inducing CD8+ cytotoxic T-cells in the tumors. Glypican-3 peptide vaccine was also evaluated as an adjuvant therapy in 41 patients with HCC after surgical resection (*n* = 35) or RFA (*n* = 6) of the primary tumor [51]. Among the 35 patients who received surgery and adjuvant glypican-3 peptide vaccine, 25 patients were found to have glypican-3 positive tumors. In the subset of patients with glypican-3 positive tumors, adjuvant glypican-3 vaccination was associated with a reduction in tumor recurrences (24% vs. 48%, *p* = 0.047). In a pre-clinical study using the samples from the above two studies, the investigators evaluated the combination of anti-PDL1 antibody and glypican-3 peptide vaccine [52]. In this study, the anti-PD-L1 antibody augmented the immune response of the vaccine [52]. In addition to the glypican-3 vaccine, a monoclonal antibody targeting the glypican-3 peptide (GC33) showed promising results in the patients harboring glypican-3 positive tumors with tolerable safety profile [53].

In contrast, other vaccines such as GV1001 (telomerase peptide vaccine) and cell-free plasmid DNA vaccine failed to show encouraging clinical response [40]. JX-594, an oncolytic pox virus vaccine demonstrated preliminary activity in a small study (*n* = 10) with a partial response, stable disease, and progressive disease in three, six, and one patient(s), respectively [54]. JX-594 is currently being evaluated in a phase III clinical trial in combination with sorafenib compared to sorafenib alone (PHOCUS trial-NCT02562755). Another phase-I/II clinical trial is currently recruiting patients with advanced HCC to evaluate the combination of JX-594 and nivolumab (NCT03071094). In addition, HEPAVAC-101 phase I/II first in-human clinical trial is planned to evaluate the role of IMA970A, a therapeutic cancer vaccine targeting tumor-associated peptides (TUMAPs) (NCT03203005) [55]. These ongoing clinical trials will hopefully provide more insight into the role of vaccine immunotherapy in HCC.

## 6. Check Point Inhibitors in HCC Management

Immune checkpoint inhibitors including tremelimumab, nivolumab, pembrolizumab, atezolizumab, and durvalumab are being evaluated for the management of HCC. Tremelimumab, a monoclonal antibody targeting CTLA4, at a dose of 15 mg/kg IV every 90 days was first evaluated in a phase II multicenter clinical trial involving twenty patients with advanced HCC from hepatitis C viral etiology [56]. About 43% of the patients had Child-Pugh class B disease. The study reported partial responses in 3 patients (~18%) while 10 patients (~60%) had stable disease with a median PFS of 6.5 months. It is important to note that the encouraging results seen in this trial occurred at the sub-optimal dosing of tremelimumab. The drug was later evaluated in combination with RFA or TACE in a phase I/II trial involving advanced HCC patients (14% were of Child-Pugh class B) [57]. Among the 19 evaluable patients, partial response was seen in five patients (26%) while 12 patients had stable disease (63%). The pathological evaluation showed increased counts of cytotoxic CD8+ T-cells in the tumor tissues. Tremelimumab is currently being evaluated either alone or in combination with another PD-1/CD-80 antibody, durvalumab (NCT02519348, NCT03298451) and liver-directed therapies (NCT02821754, NCT03482102).

Nivolumab, a monoclonal antibody against PD-1, was evaluated in an open-label multicenter clinical trial involving 154 patients with advanced HCC who progressed on sorafenib or were sorafenib intolerant (CheckMate-040) [58]. The trial included Child-Pugh class A cirrhotic patients with or without viral etiology. Among the 212 patients that received nivolumab 3 mg/kg every 2 weeks in the dose-expansion phase, 18.2% had overall response rate using modified RECIST criteria (14.3% objective response rate using RECIST 1.1 criteria). The drug was tolerated well with grade III/IV adverse events occurring in 29% and 18% of the patients in sorafenib-naïve and sorafenib-experienced groups, respectively. Nivolumab was also evaluated in 49 patients harboring advanced HCC with Child-Pugh class B in CheckMate-040 trial [59]. Among the 49 patients, 57% (*n* = 28) had extra-hepatic extension and vascular invasion. After a follow up of 7.4 months, nivolumab resulted in a disease control rate of 55% and an objective response rate of 10%. The median overall survival of the entire cohort was 7.6 months and was better in sorafenib-naïve patients (9.8 months) compared to the patients with prior sorafenib exposure (7.3 months). Given the encouraging results of this trial, nivolumab was FDA approved for management of HCC in patients who were sorafenib intolerant or had progressed on sorafenib. The FDA levied a contingency that the drug should demonstrate significant efficacy and tolerability in a large phase III trial of patients with advanced HCC (CheckMate 459-NCT02576509).

Pembrolizumab, another IgG4 monoclonal antibody targeting PD-1 receptor demonstrated encouraging preliminary results in a phase II trial while it falls short in a phase III confirmatory trial [15,60]. In the phase II trial (KEYNOTE-224), pembrolizumab was evaluated at the dose of 200 mg every 3 weeks in patients with advanced HCC (94% were of Child-Pugh class A) and previously managed with sorafenib [15]. Among 98 evaluable patients, one patient had a complete response while a partial response was seen in 17 with an objective response rate of 17%, and stable disease was seen in 46 patients. About one-fourth of the patients reported grade III/IV treatment-related adverse events and one death due to ulcerative esophagitis. Given the encouraging results, the drug was approved by the FDA in November 2018 for the use in advanced stage HCC that is sorafenib-resistant or intolerant. In February, 2019, Merck press released preliminary data of a placebo-controlled phase III trial (KEYNOTE-240) involving 413 patients previously treated with systemic therapy for HCC. The KEYNOTE-240 trial did not meet the pre-specified co-primary endpoint of statistically significant difference in median OS and PFS [60]. However, the results showed a trend favoring pembrolizumab with longer median OS (HR = 0.78; CI: 0.61–0.99) and PFS (HR = 0.78; CI: 0.61–0.99).

Another monoclonal antibody, atezolizumab, that targets PD-L1 was evaluated in combination with the anti-VEGF antibody, bevacizumab, in a phase 1b trial involving 26 treatment-naïve patients with advanced HCC [61]. Anti-VEGF therapy is known to cause immunomodulatory effects by decreasing the recruitment of CD4+ regulatory T-lymphocytes and MDSC’s while also inducing the differentiation and activation of dendritic cells [62,63]. The rationale behind the study was that the immunomodulatory changes induced by anti-VEGF therapy were thought to potentiate the effects of atezolizumab. Among the 21 evaluable patients, combination therapy resulted in a partial response in 61% (*n* = 13) of the patients. The combination was relatively well tolerated with 35% of the subjects experiencing grade III/IV adverse events. The combination is currently being evaluated in a phase III trial (NCT03434379). Table 1 summarizes the checkpoint inhibitor clinical trials in advanced HCC.

The clinical trials evaluating checkpoint inhibitors in patients with advanced HCC thus far have resulted in mixed results suggesting that there are certain groups of patients who could potentially benefit from these agents; now the challenge is the identification of those subsets of patients who are more likely to benefit. The trial that focused on nivolumab, CHECKMATE-040, evaluated PD-L1 staining, and the response rates seen occurred irrespective of the PD-L1 staining status. On the contrary, KEYNOTE-224 developed a score involving both the tumor and immune cells ratioed to the total number of viable tumor cells. The positive score had a trend for higher objective responses. The other challenge is the lack of a standard technique to measure the PD-L1 expression given the heterogeneity in PD-L1 expression and heterogeneity of HCC [64].

Multiple phase I-III clinical trials that intend to evaluate the role of checkpoint inhibitors (alone or in combination) in advanced HCC are underway (Table 1, Table 2 and Table 3). In the patients with chronic HCV infection, although immune checkpoint inhibitors that block either PD-L1/PD-1 or CTLA4 resulted in the increased expression of circulating CD8+ T-cells, the activity of intrahepatic CD8+ T-cells was unaltered. One of the plausible explanations for the refractoriness of intrahepatic CD8+ T-cells is CTLA4 upregulation and decreased activity of CD28 and CD127 on CD8+ T-cells. Interestingly, the combination of CTLA4 and PD-L1 inhibitors reversed the refractoriness of intrahepatic CD8+ T-cells indicating the synergistic effect of the combination therapy with PD-L1 and CTLA4 inhibitors [65]. The combination of ipilimumab and nivolumab is currently being evaluated as a neoadjuvant therapy in patients undergoing hepatic resection (NCT03682276, NCT03510871). In addition, checkpoint inhibitors are also currently being evaluated as adjuvant therapeutic agents in patients who have undergone curative resection of the primary tumor. For instance, the JUPITER-04 trial (NCT03859128) is planned to evaluate the role of toripalimab (anti-PD-1 antibody) as adjuvant therapy in HCC. The primary end of the study is to determine the recurrence-free survival. EMRALD-2 is another randomized phase III trial (NCT03847428) that is designed to evaluate the role of durvalumab and bevacizumab, alone or in combination in the adjuvant setting. Similarly, CheckMate 9DX (NCT03383458) is designed to evaluate the role of nivolumab in the post-resection or post-ablation adjuvant setting (Table 4). Pembrolizumab, in the study KEYNOTE-937, is being compared to placebo in a phase 3 study following curative resection or ablation, with recurrence-free survival as the primary outcome measure (NCT03867084). The results of these trials are much awaited and hopefully, these trials provide more insight on the role of immunotherapy in this dismal malignancy.

## 7. Role of Cellular Therapies in HCC

Adoptive cell transfer, chimeric antigen receptor T-cells (CAR-Ts), and T-cell receptors constitute cellular therapies in the management of HCC. Adoptive cell therapy consists of harnessing the host cytotoxic T-cells that attack the tumor-specific antigens through T-cell receptors. These T-cells that attack the tumor tissue (also known as tumor-infiltrating lymphocytes) showed promising results in certain malignancies [68,69]. A meta-analysis evaluated the role of adoptive cell therapy in the patients with HCC that were managed with surgical resection of the primary tumor. Despite the fact that adoptive cell therapy did not yield in OS benefit (odds ratio: 0.91; *p* = 0.792), the therapy resulted in significant decrease in recurrence-free survival at the end of one (odds ratio: 0.35; *p* = 0.0003) and three years (odds ratio: 0.31; *p* = 0.0001) [70]. A Chinese, non-randomized study evaluated the role of cytokine-induced killer (CIK) cells in combination with liver-directed therapies, TACE+RFA. The patients who received the combination of CIK+TACE+RFA had significantly improved OS (56 vs. 31 months; *p* = 0.001) and PFS (17 vs. 10 months; *p* = 0.001) compared to that of the group that received liver directed therapies only [71]. After the preliminary encouraging results of CIK cells in HCC, a randomized phase II trial confirmed the benefits of CIK in non-surgical HCC patients. The trial concluded that the group that received CIK along with standard treatment had significant improvement in median OS and PFS at the end of 1, 2, and 3 years [72]. A randomized phase III trial yielded similar promising results in improving the recurrence-free survival with a tolerable side effect profile [73].

A phase I trial evaluated the role of anti-glypican-3 CAR-Ts in HCC management [74]. The trial included refractory glypican-3 positive HCC tumors and evaluated the results in two groups, lymphocyte-depleted (*n* = 8) and non-lymphocyte depleted (*n* = 5) cohorts. In the lymphocyte depleted group (achieved with fludarabine and cyclophosphamide), among six evaluable patients, partial response was seen in one patient while three patients had stable disease and two had progressive disease. On the contrary, all five patients with no lymphocyte depletion had progressive disease. While this trial resulted in mixed results, it opened doors to CAR-T therapy in HCC. Results from the ongoing clinical trials evaluating the role of CAR-Ts and T-cell receptors in HCC are much awaited (Table 5).

## 8. Potential Challenges and Future Directions of Immunotherapy in HCC

Excitement in developing new therapeutic approaches in advanced HCC evolved over the last 5 years since the introduction of checkpoint inhibitors. While immunotherapy showed promising results in early clinical trials, the phase III clinical trial with pembrolizumab (KEYNOTE-240) compared to best supportive care yielded disappointing results, challenging the role of checkpoint inhibitors in HCC. Now the results of phase III trial evaluating the role of nivolumab are awaited to determine the role of checkpoint inhibitors in advanced HCC. One of the potential challenges with the use of immunotherapy in HCC is the lack of data on their safety in patients who received orthotopic liver transplantation, as there is a logical fear of transplant rejection and fatal liver failure [75]. There is a phase 1 study recruiting patients at this time in the subgroup of patients with Hepatitis B-related HCC status-post transplantation that aims to assess the safety, tolerability, and effectiveness of the HBV specific T cell receptor (HBV/TCR) redirected T cell (NCT02686372). Though there are case reports that immunotherapy was successfully used in patients that received liver transplantation, long-term safety data in HCC is lacking [76,77]. Given the heterogeneity of HCC, another challenge is identifying the sub-set of patients who are more likely to benefit from the immunotherapy. For example, Harding et al. utilized the next-generation sequencing to identify the resistance mechanisms and druggable targets, which could potentially determine the response of systemic therapy [78]. The group identified that HCC tumors harboring *Wnt/CTNNB1* mutations were refractory to immune checkpoint inhibitors. All patients harboring *Wnt/CTNNB1* (*n* = 7) and *AXIN1* (*n* = 3) mutations had progressive disease as the best response with a poor median OS. On the contrary, objective responses to checkpoint inhibitors were observed in patients with tumors harboring tumor-infiltrating lymphocytes. These studies potentially open the door for precision oncology in HCC by integrating next-generation sequencing to match HCC patients to immunotherapy.

Another subgroup affected by limited immunotherapy studies that may benefit are patients that have an altered liver function with Child-Pugh B disease. Tremelilumab, in the previously mentioned phase II trial by Sangro et al., was studied with predominantly Child-Pugh B disease (43%) and the conclusion by the research team was that it supported further investigation [56]. Another single-center retrospective study analyzed nivolumab in Child-Pugh Class B patients [79]. Of the 18 patients evaluated, two patients had partial response whereas one patient had a complete response. Nivolumab resulted in median PFS and OS of 1.6 and 5.9 months, respectively. The study reported that immune-related adverse events in class B patients were similar to that of class A patients (50%). Future studies involving a larger group of Child-Pugh class B patients will shed more light on the role of checkpoint inhibitors in this specific sub-group. 

In addition, the future of immunotherapy in HCC might rely on the combination therapy approach: either combination of two checkpoint inhibitors, or combination of checkpoint inhibitors and targeted therapy that blocks VEGFR, or a combination of checkpoint inhibitors and liver-directed therapies such as TACE and RFA. Liver-directed therapies (TACE/RFA) not only alter the local immune environment but also leads to systemic release of tumor antigens. Hence, adding immune checkpoint inhibitors to liver directed therapies may potentiate both the tumor and systemic immune response [80]. Furthermore, agents that deplete regulatory T-cells in the tumors may help in potentiating the effects of checkpoint inhibitors. For example, the combination of anti-OX40 monoclonal antibodies (which deplete regulatory T-cells) and an anti-PD-1 therapy resulted in encouraging responses in the tissues that were previously resistant to anti-PD-1 monotherapy [81,82]. Similar positive results were seen in preclinical models that evaluated anti-LAG3 antibodies or anti-TIM3 antibodies in combination with anti PD-/PD-L1 agents [83,84]. These anti-OX40, LAG3 and TIM3 antibodies act by depleting the regulatory T-cells thereby preventing the cytotoxic CD8+ exhaustion.

## 9. Conclusions

The advent of immune checkpoint inhibitors and adaptive cell therapies have revolutionized cancer therapy and preliminary trials in HCC have shown encouraging results. While the results of phase III nivolumab trial are much awaited, a number of clinical trials are currently enrolling patients to further analyze the role of immunotherapy (either alone or in combination) in a large cohort of patients with advanced HCC. Given the heterogeneous nature of HCC, integration of next-generation sequencing may hopefully provide some insight on identifying the patients who may potentially benefit from immunotherapy. 

## Figures and Tables

**Table 1:** Key clinical trials of immune checkpoint inhibitors in hepatocellular carcinoma.

Check Point Inhibitor	Study	Significance/Outcome					Ongoing Clinical Trials
		Complete Response	Partial Response	Stable Disease	Overall Survival	PFS	
Tremelimumab	Sangro et al., (*n* = 21) [56]	0%	17.6%	~59%	8.2 months	6.4 months	NCT02519348 NCT03298451
Tremelimumab + ablation	Duffy et al., (*n* = 32) [57]	--	26.3%	--	12.3 months	7.4 months	NCT02821754 NCT03482102
Nivolumab	El-Khoueiry et al., (*n* = 48) [58]	4.2%	8.3%	--	15 months	3.4 months	NCT03383458
	El-Khoueiry et al., (*n* = 214) [58]	1.4%	18.2% *	45%	83% alive at 6 months	4.1 months	
Pembrolizumab	Zhu et al., (*n* = 104) [15]	1%	16%	44%	54% alive at 1 year	5.1 months	NCT03062358 NCT02702414 NCT03163992 NCT03419481 NCT02595866
Durvalumab	Wainberg ZA, et al., (*n* = 40) [66]	0%	10.3%	23%	13.2 months	--	NCT02519348 NCT03298451
Durvalumab + tremelimumab	Kelley RK, et al., (*n* = 40) [67]	0%	15%	45%	--	--	NCT02519348 NCT03298451

**Table 2:** Selective ongoing clinical trials of combination immunotherapy agents in advanced hepatocellular carcinoma.

Targeted Pathways	Agents Evaluated	Clinical Trial Registration Number
CTLA-4 + PD-1	Nivolumab + ipilimumab	NCT03203304
		NCT03222076
GM-CSF-armed oncolytic virus + PD-1	PexaVec + Nivolumab	NCT03071094
Tumor infiltrating lymphocytes + PD-1	Tumor infiltrating lymphocytes + pembrolizumab	NCT01174121
PD-1 + tyrosine kinase inhibitor	Pembrolizumab + regorafenib	NCT03347292
	Pembrolizumab + lenvatinib	NCT03006926
	Nivolumab + cabozantinib	NCT03299946
	Nivolumab ± lenvatinib	NCT03418922
	Nivolumab + sorafenib	NCT03439891
	Pembrolizumab + sorafenib	NCT03211416
PD-L1 + tyrosine kinase inhibitor	Avelumab + regorafenib	NCT03475953
	Avelumab + axitinib	NCT03289533
PD-1 + anti-VEGF agents	Nivolumab + bevacizumab	NCT03382886
PD-L1 + anti-VEGF agents	Atezolizumab + bevacizumab	NCT02715531
	Atezolizumab + bevacizumab vs. sorafenib	NCT03434379
CTLA-4, PD-1, anti-OX40 antibody	INCAGN01949 + ipilimumab vs.	NCT03241173
	INCAGN01949 + nivolumab vs.	
	INCAGN01949 + nivolumab + ipilimumab	

CTLA4: cytotoxic T-lymphocyte-associated protein 4; PD-1: Programmed cell death protein-1; GM-CSF: Granulocyte monocyte-colony stimulating factor; PD-L1: Programmed cell death ligand-1; VEGF: Vascular endothelial growth factor.

**Table 3:** Selective ongoing clinical trials of the combination of immunotherapy agents with liver-directed therapies.

Targeted Pathways	Agents Evaluated	Clinical Trial Registration Number
Radiation + PD-1	Yttrium-90 + nivolumab	NCT03380130
		NCT03033446
		NCT02837029
	Yttrium-90 + pembrolizumab	NCT03099564
	SBRT + pembrolizumab	NCT03316872
Ischemia + PD-1	TACE + pembrolizumab	NCT03397654
	TACE + nivolumab	NCT03572582
	DEB-TACE + nivolumab	NCT03143270
	TACE + durvalumab + bevacizumab	NCT03778957
CTLA-4 + PD-L1 + radiation	Tremelimumab + durvalumab + radiation	NCT03482102
CTLA-4 + PD-L1 + ischemia	Tremelimumab + durvalumab + RFA/TACE/cryoablation	NCT02821754

PD-1: Programmed cell death protein-1; SBRT: Stereotactic body radiation therapy; TACE: Trans Arterial Chemoembolization; DEB-TACE: Drug-Eluting Bead Trans-Arterial Chemoembolization; CTLA4: cytotoxic T-lymphocyte-associated protein 4; PD-L1: Programmed cell death ligand-1; RFA: Radiofrequency Ablation.

**Table 4:** Selective ongoing clinical trials of immunotherapy agents in the adjuvant setting.

Targeted Pathways	Agents Evaluated	Clinical Trial Registration Number
PD-1 + Curative resection	Toripalimab	NCT03859128
PD-L1 + VEGF + Curative resection or ablation	Durvalumab or Durvalumab + bevacizumab	NCT03847428
PD-1 + Curative resection or ablation	Nivolumab	NCT03383458
PD-1 + Curative resection or ablation	Pembrolizumab	NCT03867084

PD-1: Programmed cell death protein-1; PD-L1: Programmed cell death ligand-1; VEGF: Vascular endothelial growth factor.

**Table 5:** Selective ongoing clinical trials of adaptive-cell therapies in hepatocellular carcinoma.

Targeted Pathways	Agents Evaluated	Clinical Trial Registration Number
Adaptive cell therapy AFP targeted T-cell receptors	Autologous genetically modified AFPᶜ^332^T cells	NCT03132792
	Autologous ET1402L1-CART cells	NCT03349255
Glypican-3 antigen	CAR-Glypican3 T cells	NCT03146234 NCT03198546 NCT02715362 NCT03130712 NCT02959151
	Glypican 3-specific Chimeric Antigen Receptor Expressing T Cells for Hepatocellular Carcinoma (GLYCAR) (GLYCAR)	NCT02905188
Glypican-3 antigen	CAR-Glypican3 T cells; leukocyte depletion with cyclophosphamide and fludarabine	NCT03084380 * NCT03302403 ^^,^*
c-Met/PD-L1	c-Met/PD-L1 CAR-T Cell Injection	NCT03672305 *

AFP: Alfa fetoprotein; CAR-T: Chimeric Antigen Receptor T-cells; c-Met: Tyrosine kinase MET; PD-L1: Programmed Cell Death Ligand 1. ^ Basket trial; * Not recruiting patients yet.
